# Crossroads: the role of biomarkers in the management of lumps in the breast

**DOI:** 10.18632/oncotarget.28402

**Published:** 2023-04-24

**Authors:** Georg F. Weber

**Affiliations:** ^1^University of Cincinnati Academic Health Center, Cincinnati, OH 45267, USA

**Keywords:** breast cancer premalignant lesion, biomarker, biopsy, mammography

## Abstract

Premalignant lesions in the breast pose a difficult decision-making problem, whether to treat proactively and accept the side effects or to engage in watchful waiting and possibly encounter a later diagnosis of invasive cancer. A biomarker or set of biomarkers to inform on the individual progression risk would be beneficial to the patient and cost-effective for the healthcare system. The gene products of tumor progression may be expressed in early non-cancerous (“premalignant”) lesions, where they are associated with a high probability for full transformation in breast cancers. One such molecule is the OPN splice variant-c. OPN-c is also present in a fraction of the premalignant lesions, where it reflects an elevated risk for progression to cancer within 5 years, regardless of the lesion’s subtype. This marker has the properties needed to facilitate decisions to treat at the premalignant stage.

## INTRODUCTION

Over almost 80 years since the development of the first effective cancer chemotherapies in 1946, disease management has undergone several historic changes [1]. The principal philosophies of cancer care have evolved from initially increasingly aggressive treatments to more measured responses. Further, the focus has shifted to emphasize prevention and early detection, so as to avoid the still challenging treatment of an established cancer. In the development of applicable prevention and detection regimens, tumors of the breast have been at the forefront because of the accessibility of the organ to diagnostic procedures. Mammography and biopsies have become standard practice.

With these measures, a new problem has arisen. Women over the age of 40 years often have lumps in their breasts that are not cancerous at the time of biopsy (comprising atypias, hyperplasias, papillomas, radial scars, lobular carcinoma *in situ*, ductal carcinoma *in situ* (DCIS)). These patients need to make the difficult decision whether to treat the lesions proactively and accept the substantial compromise in their quality of life (from surgery, radiation, or hormone therapy: Surgery often ensues for definitive diagnosis. Radiation may follow the surgical resection of DCIS by lumpectomy. Hormone therapy can come into play as a strategy for risk reduction if the estimated 5-year risk exceeds 1.6% in the Gail model [2]) or to engage in watchful waiting and risk a later diagnosis of invasive cancer (the proverbial sword of Damocles) [3–7]. Currently, two forms of assessment are available to facilitate making that choice:

- Each type of lesion is associated with broad-range estimates for progression risk. However, such prognostications of the likelihood for developing cancer are not very useful when they are provided in wide brackets, such as 30–50% for DCIS.
- Inspection of the lesion’s margins for microinvasion is informative but requires step sections through the biopsied tissue. The evaluation is tedious and prone to false negative results. Further, suboptimal breast localization during the procedure can cause compromised margins of the lesion to be missed.

A biomarker or set of biomarkers to inform on the individual progression risk would be beneficial to the patient and cost-effective for the healthcare system. (At 1.6 million biopsies per year in the US, the presumed overestimation of risk in 24% of patients leads to overtreatment of 384,000 patients per year at a cost of $61,000/patient. The presumed underestimation of risk in 21% of DCIS cases leads to missed intervention in 336,000 patients, who may return as stage 4 patients and require treatment at $135,000/year [8–10]). With a marker-based approach, relevant information is obtainable from one stained slide, not requiring step sections. Also, a less than perfect breast localization during biopsy is not detrimental to the analysis of tissue staining.

Biomarkers are either prognostic (inform on the natural course of the disease) or predictive (inform on prospective responses to treatment). Their application is useful only if the results are actionable in the clinic. The oncology literature is full of descriptions pertaining to biomarkers that associate with survival or grade. They have no practical bearing. Grade can be assessed by a pathologist without the use of markers, and the assignment to a high or low survival subgroup of an existing cancer very rarely changes the applied treatment regimen. By contrast, the prognostication of progression risk for an individual patient diagnosed with a premalignant lesion can be eminently meaningful, as it facilitates the decision whether (and how broadly) to treat preemptively or to engage in watchful waiting.

In this regard, OPN splice variants have proven useful. The cytokine OPN (short for the misnomer “Osteopontin”) has long been associated with the progression of several types of cancer, including those of the breast [11]. Unfortunately, the extensive posttranslational modifications of the molecule together with its physiologic role in cellular immunity and its secretion into the breast milk for calcium binding have limited the biomarker potential of OPN in cancer, and specifically in breast cancer. The discovery that – beside the full-length form – alternatively spliced, shorter versions are produced by transformed cells [12] has opened the door for novel biomarker development. Most breast cancers produce the full-length form (OPN-a) together with the shortest splice variant (OPN-c). Remarkably, OPN-c is also present in a fraction of the premalignant lesions, where it reflects a high risk for progression to cancer within 5 years, regardless of the lesion’s subtype [13, 14]. With the simple immunohistochemical staining of the biopsied material, the individualized progression risk for that patient can be estimated with good sensitivity and specificity (Table 1), and it can lead to improved counseling.

**Table 1:** OPN splice specificity and sensitivity in premalignant lesions

Healthy breasts, hyperplasias, papillomas, and carcinomas *in situ* from 434 women [13]. ~10% of OPN-c pathology score 0–1 (intensity), vs. 40% of score 3 experience cancer over 5 years. >90% of women, who progress, had pathology scores of 2–3 for OPN-c intensity at the time of initial diagnosis. Combining OPN-c and OPN-exon-4 staining → all low intensity patients are alive after 5 years, women in the high category have a close to 30% chance to die. Of patients who succumb, close to 80% had a high combined score at initial diagnosis.
Papillomas from 114 women [14]. <5% of OPN-c pathology score 0–1 (intensity) versus almost 18% of score 2–3 experience cancer in follow-up. 9 of 12 women (75%), who progressed, had pathology scores of 2–3 for OPN-c intensity at the time of initial diagnosis, none had a score of 0. Combined risk score from intensity plus percent positivity for OPN-c → progression risk for low score = 3.2%, intermediate score = 5.7%, high score = 18.8% (RR 4.043, CI 95% 1.159–14.109). 6 patients later diagnosed with cancer in the contralateral breast → high OPN-c staining in >80%. Combined score from OPN-c → contralateral progression risk for low score = 3.0%, intermediate score = 0%, high score = 10.0% (RR 7.143, CI 95% 0.866–58.946). Substantially reduced fraction of low scores in OPN-exon-4 for later cancer in the contralateral breast.

Circumstantial support comes from the observation of total OPN (4 studies, 172 patients [18]) or OPN-c (3 studies, 45 patients [19]) in the progression of premalignant lesions, and from increasing OPN-c blood levels with DCIS progression (67 patients [20]).

Frequently, marker combinations have been found to be more informative than individual markers. In fact, panels have been developed for the evaluation of various breast conditions (Table 2), but the risk prediction for premalignant lesions has not yet been covered. There is opportunity for the development of panels that will aid women with such mammary lumps in deciding on how to proceed. The prognostication of progression risk with OPN-c may be combined with OPN exon 4 to evaluate survival prospects [13]. The proliferation marker Ki-67 may serve as an additional readout for lesion aggressiveness [15]. ER, PR, and HER2 inform on treatment choices if the decision is made in favor of preemptive action. Additional combinations are conceivable.

**Table 2:** Diagnostic devices for mammary transformation

Test	Description	Purpose	Target population
*DCISionRT*	Lesion size, patient age, IHC:HER2, PR, Ki67, COX2, p16/INK4A, FOXA1, SIAH2	Calculate 10-year local recurrence risk	DCIS, decision on radiation therapy
*Oncotype DX Test*	Expression levels of 12 genes	Recurrence/progression risk	DCIS
*Oncotype DX Test*	Expression levels of 12 genes	Benefit from chemotherapy after surgery	DCIS
*Oncotype DX Test*	Expression levels of 21 genes	Recurrence risk	Early-stage (1–2) ER positive breast cancer, LN negative
*Oncotype DX Test*	Expression levels of 21 genes	Benefit from chemotherapy after surgery	Early-stage (1–2) ER positive breast cancer, LN negative
*MammaPrint*	Amsterdam 70-gene breast cancer gene signature	Benefit from chemotherapy	Early stage (1–2) breast cancer patients, tumor <5 cm
*MammaPrint*	Amsterdam 70-gene breast cancer gene signature	Metastasis risk (high/low)	Early stage (1–2) breast cancer patients, tumor <5 cm
*Prosigna*	Gene expression signature for 58 genes	Treatment decisions based on the risk of distant recurrence	Early-stage (1–2), hormone-receptor-positive breast cancer (post-surgery/hormone)
*Prosigna*	Gene expression signature for 58 genes	Treatment decisions based on the risk of distant recurrence	Stage 2, <4 LN positive, hormone-receptor-positive breast cancer (post-surgery/hormone)
*EndoPredict*	Lesion size, LN involvement, UBE2C, BIRC5, DHCR7, STC2, AZGP1, IL6ST, RBBP8, MGP, 4 control genes	Risk for distant metastases, chemotherapy decision	Newly diagnosed, early-stage (1–2), ER positive, HER2 negative breast cancer
*Mammostrat*	P53, HTF9C, CEACAM5, NDRG1, SLC7A5 (IHC)	Risk of metastasis and recurrence	Newly diagnosed, early stage breast cancer
*BRCANext*	18 genes associated with hereditary breast cancer	Consideration of prophylactic mastectomy	Suspicion of hereditary predisposition
*LobSig*	194-gene set	Outcome prediction	Invasive lobular carcinoma
*GGI/MapQuantDx*	97 genes involved in cell cycle regulation or proliferation	Prognostic marker and predictor of outcome	Tamoxifen-treated patients
*Blueprint*	80-gene signature	Breast cancer subtyping	Breast cancer
*Breast Cancer Index Test*	Molecular Grade Index, Genes: BUB1B, CENPA, NEK2, RACGAP1, RRM2, HoxB13, IL17BR	5–10 Year recurrence risk, decision on extending hormone therapy	LN negative, hormone-receptor-positive, HER2 negative breast cancer, post-hormone treatment
*Guardant 360*	Liquid biopsy, ALK, ATM, BRAF, BRAF V600E, BRAF V600K, BRCA1, BRCA2, CDK12, EGFR, ERBB2 (HER2), ESR1, FGFR2, FGFR3, IDH1, KIT, KRAS, MET, MSI, NRAS, NTRK, PDGFRA, PIK3CA, RET, ROS1	Treatment decisions	Advanced stage

Currently available tests are described for their basis, their purpose, and the target population they serve.

The use of molecular markers in tissue staining, even though semi-quantitative when expressed as a pathology score, is likely more accurate than scores based on macroscopic variables, such as the Van Nuys Prognostic Index (tumor size, margin width, pathologic classification, and age). This index represents a score for predicting the risk of local recurrence in patients with DCIS [16, 17]. It has limited discrimination and its strongest component is the margin width after surgical resection. Biomarkers, by contrast, are applicable to biopsies without surgery.

## CONCLUSION

Decades of cancer diagnosis and treatment have achieved substantial improvements. Yet, with every milestone of progress, new needs have surfaced. Breast care is privileged to have the availability of mammography and biopsy to assess the propensities of lumps. A meaningful next step needs to entail biomarker development, pointing the way toward either preemptive treatment or watchful waiting at the crossroad.
